# Long-Term Prognostic Value of Restitution Slope in Patients with Ischemic and Dilated Cardiomyopathies

**DOI:** 10.1371/journal.pone.0054768

**Published:** 2013-01-18

**Authors:** Marc Dorenkamp, Andreas J. Morguet, Christian Sticherling, Steffen Behrens, Markus Zabel

**Affiliations:** 1 Department of Cardiology, Charité – Universitätsmedizin Berlin, Campus Virchow-Klinikum, Berlin, Germany; 2 Department of Cardiology and Pulmonology, Charité – Universitätsmedizin Berlin, Campus Benjamin Franklin, Berlin, Germany; 3 Division of Cardiology, University Hospital, Basel, Switzerland; 4 Department of Cardiology, Vivantes Humboldt-Klinikum, Berlin, Germany; 5 Department of Cardiology and Pneumology, Heart Center, Georg-August-University of Göttingen, Göttingen, Germany; University of Illinois at Chicago, United States of America

## Abstract

**Background:**

An action potential duration (APD) restitution curve with a steep slope ≥1 has been associated with increased susceptibility for malignant ventricular arrhythmias. We aimed to evaluate the “restitution hypothesis” and tested ventricular APD restitution slope as well as effective refractory period (ERP)/APD ratio for long-term prognostic value in patients with ischemic (ICM) or dilated cardiomyopathy (DCM).

**Methodology/Principal Findings:**

Monophasic action potentials were recorded in patients with ICM (n = 32) and DCM (n = 42) undergoing routine programmed ventricular stimulation (PVS). Left ventricular ejection fraction was 32±7% and 28±9%, respectively. APD and ERP were measured at baseline stimulation (S_1_) and upon introduction of one to three extrastimuli (S_2_–S_4_). ERP/APD ratios and the APD restitution curve were calculated and the maximum restitution slope was determined. After a mean follow-up of 6.1±3.0 years, the combined end-point of mortality and and/or implantable cardioverter-defibrillator shock was not predicted by restitution slope or ERP/APD ratios. Comparing S_2_ vs. S_3_ vs. S_4_ extrastimuli for restitution slope (1.5±0.6 vs. 1.4±0.4 vs. 1.3±0.5; *p* = NS), additional extrastimuli did not lead to a steepening restitution slope. ERP/APD ratio decreased with additional extrastimuli (0.98±0.09 [S_1_] vs. 0.97±0.10 [S_2_] vs. 0.93±0.11 [S_3_]; *p* = 0.03 S_1_ vs. S_3_). Positive PVS was strongly predictive of outcome (*p* = 0.006).

**Conclusions/Significance:**

Neither ventricular APD restitution slope nor ERP/APD ratios predict outcome in patients with ICM or DCM.

## Introduction

In developed countries, sudden cardiac death (SCD) significantly contributes to cardiovascular mortality [1]. Major causes for SCD are fast ventricular tachycardia (VT) or ventricular fibrillation (VF). Findings from computer simulations and experimental studies have suggested that VF occurs when electrical waves break up into multiple re-entrant wavelets and eventually disintegrate into completely irregular excitation [2], [3], [4], [5], [6].

Among other mechanisms, an electrical restitution characterized by a steep slope of the restitution curve (≥1) may directly promote wavebreaks [7], [8], [9]. Restitution refers to the relation of action potential duration (APD) to its preceding diastolic interval (DI), graphically defining the so-called APD restitution curve. Due to its potential importance this arrhythmia mechanism has been named the “restitution hypothesis” [9]. Effective refractory period (ERP), absolute and relative to APD, is another major determinant of electrical tissue properties. In this respect, small ERP/APD ratios have been demonstrated to favor re-entrant ventricular arrhythmias but their clinical prognostic value has never been tested. Human studies on the usefulness of measuring restitution slopes have produced equivocal findings [10], [11], [12], [13].

We therefore set out to evaluate the potential clinical application of the “restitution hypothesis” in patients with ischemic cardiomyopathy (ICM) or dilated cardiomyopathy (DCM) and tested ventricular APD restitution slope and ERP/APD ratio as long-term predictors of outcome [9]. For the first time, restitution parameters of PVS with two and three extrastimuli were also calculated.

## Materials and Methods

### Patients

Seventy-four patients with ICM (n = 32) and DCM (n = 42) were prospectively enrolled into a single-center observational study at the Charité University Hospital, Campus Benjamin Franklin, Berlin, Germany, between April 1999 and August 2004. All patients had a clinical indication for electrophysiological (EP) testing including suspected arrhythmogenic syncope, documented sustained VT, or non-sustained VT and a reduced LV ejection fraction (LVEF) ≤40%. Patients with valvular disease, pacemaker or implantable cardioverter-defibrillator (ICD) at the time of the EP study were excluded. Antiarrhythmic medications were discontinued for at least 5 half-lives before the procedure. In the ICM group, patients with an acute coronary syndrome within the preceding 30 days or coronary revascularization within 6 weeks were also excluded. All patients had undergone routine clinical evaluation including echocardiography, coronary and left ventricular angiography. In a subset of 26/74 (35%) patients, microvolt T wave alternans (TWA) was measured during incremental exercise using a Cambridge Heart CH 2000 system (Cambridge Heart Inc., Bedford, MA, USA). TWA tests were classified according to previously published criteria [14]. The study protocol was approved by the Institutional Review Board of the Charité – Universitätsmedizin Berlin (Reference no. EA4/082/12) and written informed consent was obtained from all patients prior to the procedures.

### Electrophysiological study and pacing protocols

Under fluoroscopic guidance, standard multielectrode EP catheters were placed in the high right atrium and in the His bundle region. A fractally coated iridium electrode catheter (7F) (MAPCath, Biotronik, Berlin, Germany) was introduced into the right ventricle [15]. Endocardial monophasic action potentials (MAPs) were generally recorded from the right ventricular apex (RVA). In a subgroup of patients (n = 23) MAPs were also recorded from the right ventricular outflow tract (RVOT). All electrocardiographic signals were displayed and recorded on a BARD electrophysiology system (BARD LabSystem, Lowell, MA, USA). The sampling rate was 1 kHz and MAPs were being recorded with a filter bandwidth from 0.05 to 500 Hz.

In order to determine APD at 90% repolarization (APD_90_) at different heart rates, constant baseline pacing (S_1_) was performed for 30 seconds at basic cycle lengths (BCL) of 600, 500, 400, and 330 ms, followed by a pause of 30 seconds. An accelerated protocol for programmed ventricular stimulation (PVS) was performed at the above cycle lengths from both RVA and RVOT [16]. Three extrastimuli were delivered in late diastole and coupling intervals were reduced in decrements of 10 ms until refractoriness or a minimum of 200 ms. MAP recordings were obtained during PVS at a BCL of 500 ms. Thereby, the first extrastimulus (S_2_) was delivered after a drive train of eight S_1_ stimuli (BCL 500 ms) [10], [17]. Recordings were repeated with progressively shorter S_1_–S_2_ intervals (by 20 ms from 500 to 400 ms, by 10 ms from 400 to 300 ms, and by 5 ms from 300 ms to ventricular refractoriness). ERP was defined as the longest S_1_–S_2_ interval that failed to capture the ventricle. The S_1_–S_2_ interval was then increased by 10 ms to restore ventricular capture and a second extrastimulus (S_3_) was introduced at a 500-ms delay from the preceding S_2_ response repolarization. The S_2_–S_3_ interval was then decreased until refractoriness and determination of the shortest interval with ventricular capture. Finally, a third extrastimulus (S_4_) was introduced in the same manner. To determine the ERP associated with the last (S_4_) extrastimulus would have required the introduction of a fourth (S_5_) extrastimulus which was not part of the protocol. PVS was considered positive in case of reproducible induction of sustained monomorphic VT with up to 3 extrastimuli, or in case of polymorphic VT, ventricular fibrillation and/or flutter with up to 2 extrastimuli [16], [18].

### Analysis of MAP recordings

Recordings were analyzed offline using customized and validated software (National Instruments, Austin, TX, USA) [19]. MAPs were evaluated at four different BCL (S_1_) and after introduction of extrastimuli (S_2_–S_4_). APD_90_ was defined as the interval between MAP onset and 90% repolarization. The DI was calculated as the interval of time from the preceding APD_90_ to the onset of the next MAP. APD restitution curves were generated for each extrastimulus (S_2_–S_4_) by plotting APD_90_ versus preceding DI. Maximum slopes were determined by fitting the data with overlapping least-squares linear segments as previously described [12], [13]. Briefly, the restitution curves were analyzed in 40-ms DI segments in steps of 10 ms, beginning with the shortest DI range containing data (e.g. from 0 to 40 ms, then from 10 to 50 ms, 20 to 60 ms and so on). No extrapolation was performed. ERP was analyzed for basic electric stimuli (S_1_) and the first two extrastimuli (S_2_ and S_3_). The relation between ERP and APD_90_ (ERP/APD_90_ ratio) was calculated, respectively [20].

### Long-term prospective follow-up

All patients were followed prospectively for a mean (± SD) duration of 6.1±3.0 (median 5.7, interquartile range 3.8 to 9.2) years beginning at the time of EP study until May 2009. Follow-up included review of all medical records, telephone questionnaires and information on deceased patients obtained systematically from the Berlin authorities. If an ICD was implanted during the course of the study, routine ICD interrogations were reviewed. ICD shocks were graded as appropriate or inappropriate. The combined end-point was predefined as all-cause mortality and/or appropriate ICD therapy.

### Statistical analysis

Continuous data are presented as mean ± SD and categorical data are given as frequencies. Comparisons were performed with the 2-tailed Student's *t* test for continuous variables or the Chi-square test or Fisher's exact test for categorical variables (depending on field values). Where appropriate for multiple group comparisons, a one-way ANOVA with additional Tukey's test for subcomparisons was chosen. Prognostic values were assessed using Kaplan-Meier probabilities for event free survival. Dichotomized patient groups were compared using the log-rank method. Statistical analyses were performed with SPSS for Windows (Version 16.0, SPSS Inc., Chicago, IL, USA). A value of *p*<0.05 was considered statistically significant.

## Results

### Clinical findings

Patients of the two groups were predominantly male and had comparable LVEFs (ICM: 32±7%; DCM: 28±9%; *p* = 0.06) (Table 1). When compared with the DCM group, patients of the ICM group were significantly older. Digoxin use was significantly more frequent in patients with DCM. In addition to the pre-existing medication, amiodarone therapy was initiated later than the EP recordings in 15 patients (47%) with ICM and in five patients (12%) with DCM. Of the 26 TWA patients, 17 (65%) were graded positive, 7 (27%) negative, and 2 (8%) indeterminate. Positive and indeterminate tests were grouped as non-negative.

**Table 1:** Baseline clinical characteristics.

	ICM	DCM	
Clinical parameter	(n = 32)	(n = 42)	*p*
Age, y	65±9	48±12	<0.0001
Male sex, n (%)	25 (78)	34 (81)	0.78
LVEF, %	32±7	28±9	0.06
Beta-blockers, n (%)	31 (97)	41 (98)	0.90
ACE inhibitors/ARB, n (%)	32 (100)	41 (98)	0.60
Spironolactone, n (%)	22 (69)	35 (83)	0.16
Digoxin, n (%)	6 (19)	40 (95)	<0.0001
Diuretics, n (%)	30 (93)	33 (79)	0.07
Amiodarone, n (%)	2 (6)	2 (5)	0.78
Positive PVS, n (%)	13 (41)	9 (21)	0.07
ICD implantation (post MAP), n (%)	19 (59%)	4 (10%)	<0.0001

ACE  =  angiotensin-converting enzyme, ARB  =  angiotensin-receptor blocker, ICD  =  implantable cardioverter-defibrillator, MAP  =  monophasic action potential recording, PVS  =  programmed ventricular stimulation.

### Inducibility at PVS and ICD treatment

Sustained ventricular arrhythmias were inducible in 22/74 patients (30%) (Table 1). Subsequent prophylactic ICD implantation was performed in 12/13 (92%) of inducible and in 7 of non-inducible ICM patients. In the DCM group, a total of 4 patients underwent ICD implantation, 3 of them were inducible. Eventually, therapy with amiodarone was administered to 16/19 (84%) of ICD patients with ICM and 2/4 (50%) with DCM. Restitution slopes for S_2_ (1.41±0.65 vs. 1.50±0.53; *p* = 0.51), S_3_ (1.34±0.40 vs. 1.43±0.48; *p* = 0.44) and S_4_ (1.36±0.57 vs. 1.28±0.53; *p* = 0.60) did not differ between inducible and non-noninducible patients and there were no differences with respect to APD_90_ or ERP/APD_90_.

### Baseline pacing

**Figure 1:**
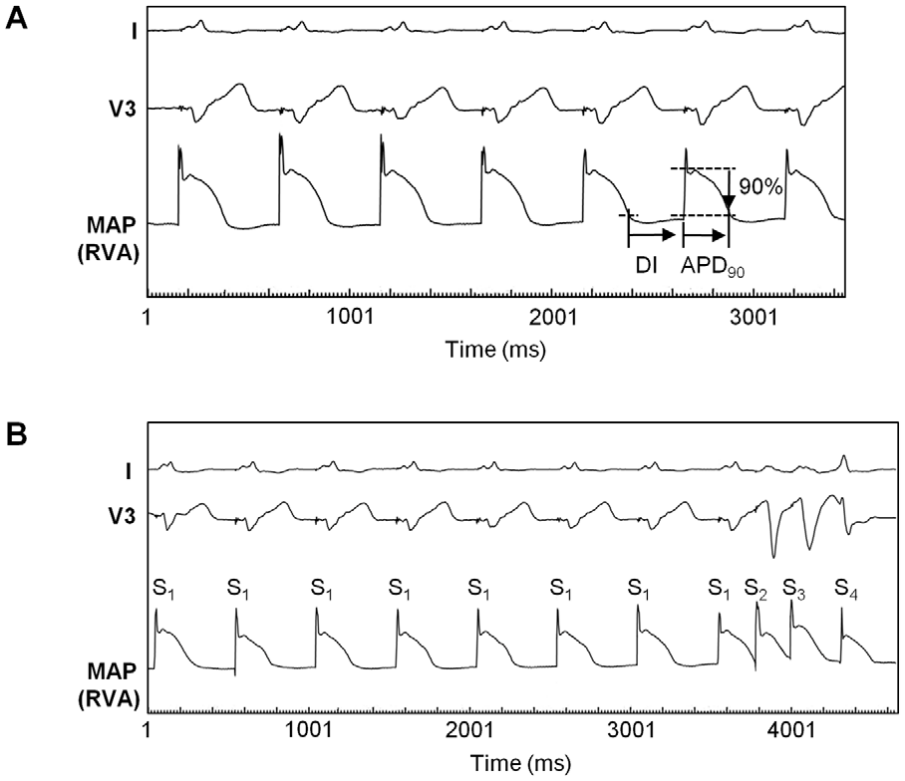
Representative MAP recordings. Monophasic action potentials (MAPs) recorded at the right ventricular apex (RVA) in a patient with ICM during baseline pacing (A) and during programmed ventricular stimulation (PVS) (B). Basic cycle length (S_1_–S_1_) was 500 ms, respectively. (A) Action potential durations (APD) were measured from MAP onset to the 90% repolarization level (APD_90_). Diastolic interval (DI) span from APD_90_ of the preceding MAP to the onset of the current MAP. (B) MAP recordings were obtained during PVS using three extrastimuli. In this example, the first two extrastimuli (S_2_ and S_3_) were already delivered at the shortest coupling intervals (S_1_–S_2_ 235 ms, S_2_–S_3_ 218 ms), while the introduction of the third extrastimulus (S_4_) was still in progress and the shortest possible S_3_–S_4_ interval had not been reached yet.

APD_90_ was prolonged along with the increase in BCL (274±42 ms [600 ms] vs. 258±35 ms [500 ms] vs. 237±29 ms [400 ms] vs. 219±24 ms [330 ms]; *p*<0.05 respectively). No significant differences could be found between the 2 recording sites (i.e. RVA vs. RVOT) or patient groups (i.e. ICM vs. DCM) with respect to all 4 BCLs. Figure 1A illustrates ventricular MAPs during baseline pacing at a BCL of 500 ms.

### Restitution slope of APD_90_

**Figure 2:**
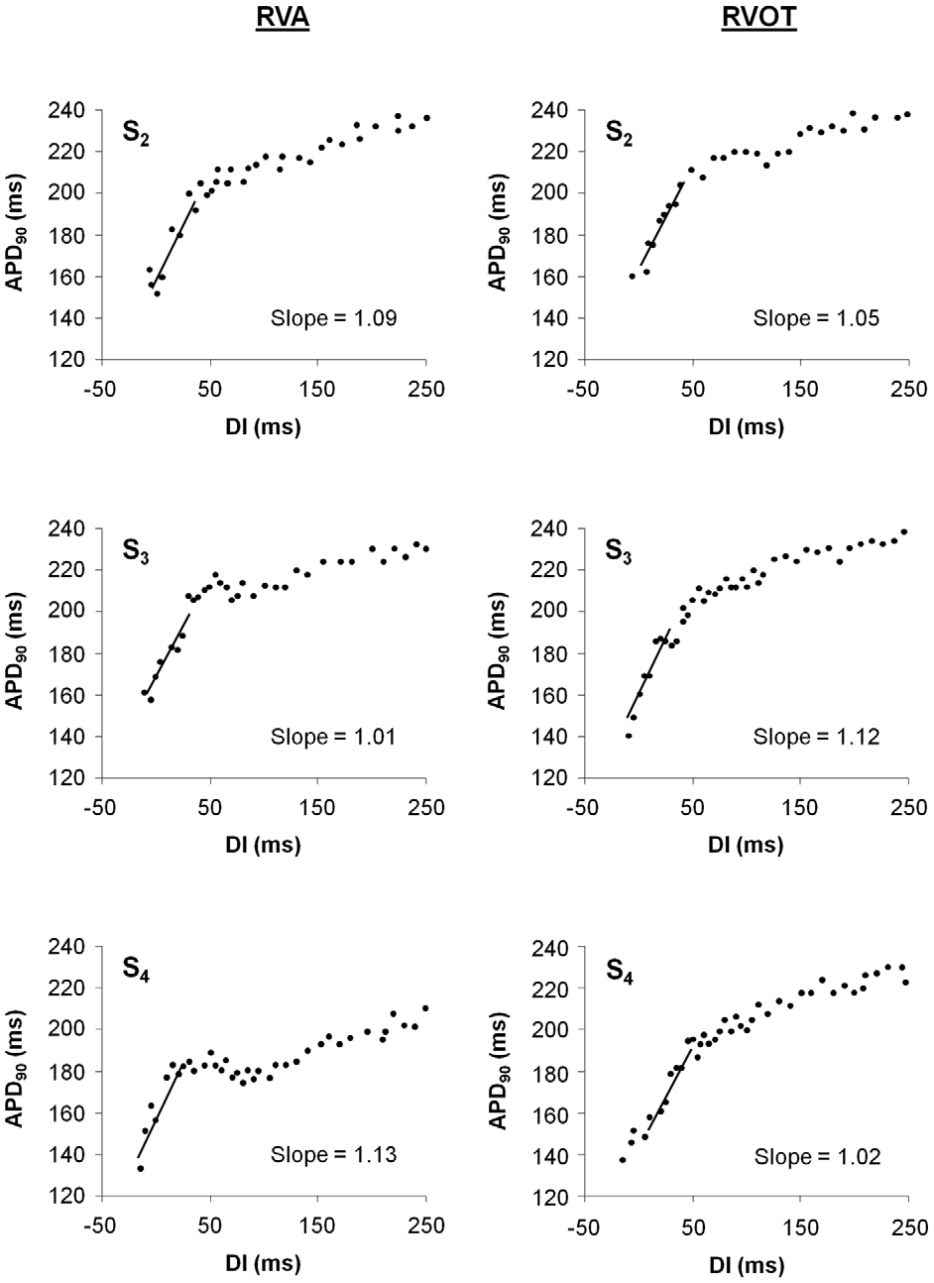
Representative restitution curves. APD_90_ restitution curves from one patient with DCM recorded at the right ventricular apex (RVA) and the right ventricular outflow tract (RVOT). For both recording sites, the restitution curves of S_2_, S_3_, and S_4_ are shown. The linear fit to the 40 ms diastolic interval (DI) with the maximum slope is superimposed on the respective curve. The maximum slope value is denoted adjacent to the curve.

Figure 1B shows a representative example of MAP recordings during PVS using three extrastimuli (S_2_–S_4_). A total of 282 APD_90_ restitution curves were constructed. A complete set of APD_90_ restitution curves from a stimulation site consisted of 3 curves each (S_2_, S_3_, and S_4_). Complete evaluation of three restitution curves (one set) originating from the RVA could be accomplished in all 74 study patients. At the RVOT, only 5 sets (16%) were analyzable in the ICM group and 15 sets (36%) in the DCM group (Table 2) due to instability of signals and catheter. Figure 2 shows an example of six APD_90_ restitution curves in a given patient (two sets). Regression lines for the steepest segment are superimposed revealing a maximum slope ≥1 in each of the 6 curves. Maximum APD_90_ restitution slopes did not differ significantly between patients with ICM and those with DCM and there were no significant differences between RVA and RVOT (Table 2). The prevalence of maximum slope ≥1 was similar (mean average prevalence of 78%) among both groups with no significant differences between the 2 recording sites or the 3 extrastimuli. No MAP alternans was observed in any of the study patients. Restitution slopes for S_2_ (1.42±0.57 vs. 1.68±0.39; *p* = 0.29), S_3_ (1.39±0.62 vs. 1.59±0.44; *p* = 0.47) and S_4_ (1.37±0.66 vs. 1.36±0.36; *p* = 0.97) did not differ between non-negative and negative TWA patients. There were no differences for S_2_ (1.50±0.66 vs. 1.46±0.51; *p* = 0.79), S_3_ (1.34±0.43 vs. 1.44±0.47; *p* = 0.37), and S_4_ (1.27±0.50 vs. 1.32±0.56; *p* = 0.72) between all patients who received and those who did not receive amiodarone.

**Table 2:** APD_90_ restitution slope characteristics.

		ICM	DCM	
Parameter	Location	(n = 32)	(n = 42)	*p*
No. with MAP recordings, n (%)	RVA	32 (100)	42 (100)	
	RVOT	5 (16)	15 (36)	
Slope maximum S_2_	RVA	1.50±0.66	1.43±0.46	0.99
	RVOT	1.54±0.75	1.39±0.62	0.69
Slope maximum S_3_	RVA	1.32±0.33	1.49±0.53	0.73
	RVOT	1.54±0.39	1.52±0.43	0.93
Slope maximum S_4_	RVA	1.27±0.46	1.32±0.59	0.66
	RVOT	1.55±0.63	1.38±0.4	0.50
No. with max. slope S_2_≥1, n (%)	RVA	25 (78)	33 (79)	0.96
	RVOT	4 (80)	10 (67)	0.56
No. with max. slope S_3_≥1, n (%)	RVA	25 (78)	34 (81)	0.76
	RVOT	5 (100)	12 (80)	0.28
No. with max. slope S_4_≥1, n (%)	RVA	22 (69)	27 (64)	0.60
	RVOT	4 (80)	13 (85)	0.75

RVA  =  right ventricular apex, RVOT  =  right ventricular outflow tract.

### ERP/APD ratio during programmed extrastimulation

Repetitive extrastimulation progressively decreased the ratio between ERP and APD_90_ (0.98±0.09 [S_1_] vs. 0.97±0.10 [S_2_] vs. 0.93±0.11 [S_3_]; *p* = 0.03 S_1_ vs. S_3_). With regard to the observed ERP/APD_90_ shortening effect there were no significant changes between the two patient groups or the two RV recording sites (Table 3). No differences between non-negative and negative TWA patients were observed regarding the ERP/APD_90_ ratio of S_1_ (0.94±0.04 vs. 0.96±0.02; *p* = 0.27), S_2_ (0.95±0.1 vs. 0.96±0.02; *p* = 0.69) and S_3_ (0.92±0.13 vs. 0.94±0.05; *p* = 0.67). Only a weak to moderate correlation was found between APD_90_ restitution slope maxima and ERP/APD_90_ ratios (slope S_2_ and ERP/APD_90_ ratio S_1_: *r* = −0.51; slope S_3_ and ERP/APD_90_ ratio S_2_: *r* = −0.43; slope S_4_ and ERP/APD_90_ ratio S_3_: *r* = −0.46; *p*<0.001 respectively).

**Table 3:** ERP/APD_90_ ratios at the two RV sites.

		ICM	DCM	
Parameter	Location	(n = 32)	(n = 42)	*p*
No. with MAP recordings, n (%)	RVA	32 (100)	42 (100)	
	RVOT	5 (16)	15 (36)	
ERP/APD_90_ ratio S_1_	RVA	0.99±0.10	0.97±0.09	0.87
	RVOT	0.98±0.09	0.96±0.05	0.68
ERP/APD_90_ ratio S_2_	RVA	0.97±0.13	0.96±0.08	0.99
	RVOT	0.94±0.07	0.92±0.08	0.89
ERP/APD_90_ ratio S_3_	RVA	0.95±0.13	0.92±0.11	0.84
	RVOT	0.90±0.14	0.90±0.13	0.98

RVA  =  right ventricular apex, RVOT  =  right ventricular outflow tract.

### Relationship of MAP derived parameters to outcome

**Figure 3:**
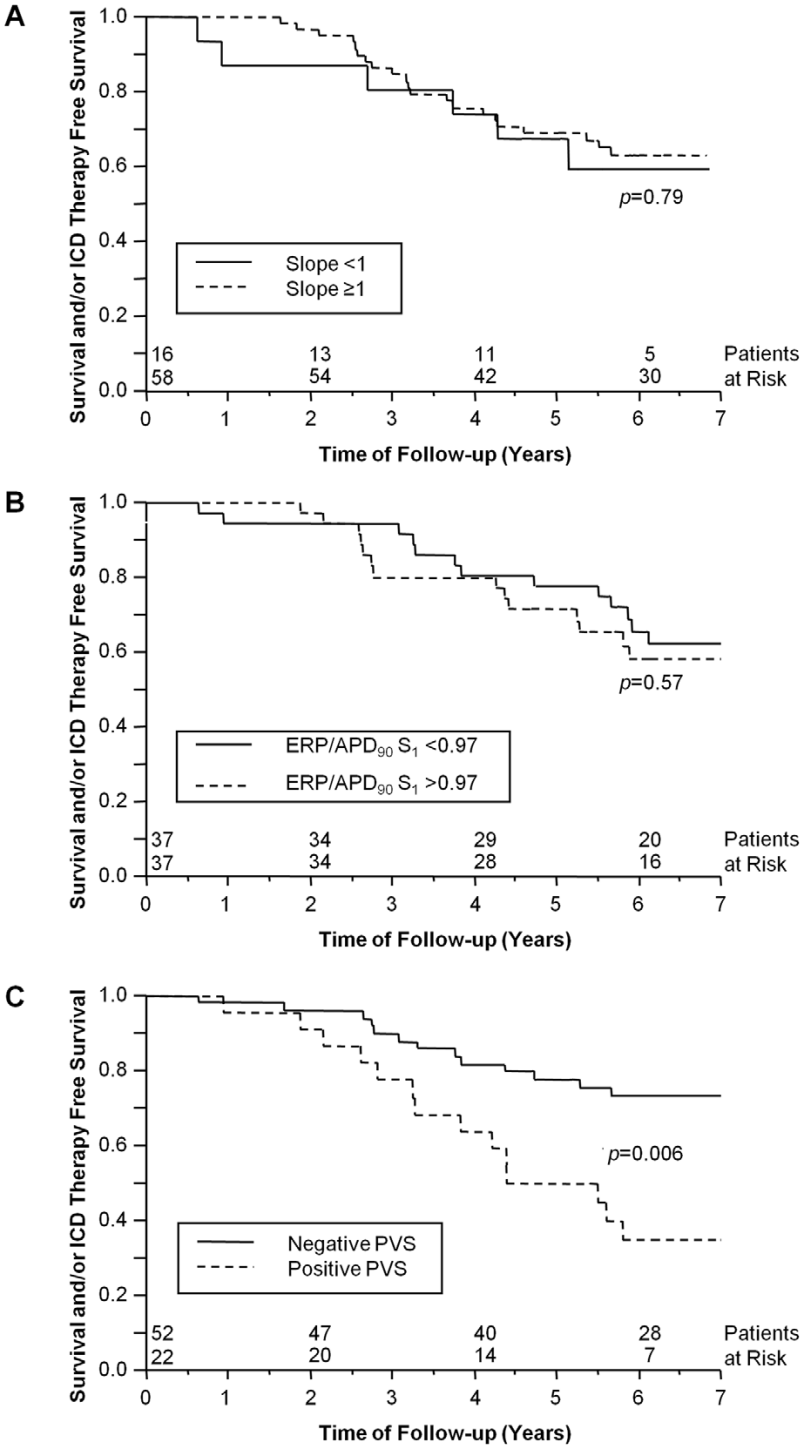
Survival curves. Kaplan-Meier survival curves for event-free survival of 74 patients with ischemic and dilated cardiomyopathy. (A) Based on maximum APD_90_ restitution slope S_2_<1 or ≥1, there was no difference in reaching the combined end-point of death and/or appropriate ICD (implantable cardioverter-defibrillator) therapy (*p* = 0.79). (B) Based on dichotomized ERP/APD_90_ ratios for S_1_, there was no difference in reaching the combined end-point of death and/or appropriate ICD therapy (*p* = 0.57). (C) Kaplan-Meier survival curves based on negative or positive programmed ventricular stimulation (PVS). Mortality and/or appropriate ICD therapy was higher in patients with positive PVS (*p* = 0.006).

Of the 74 study patients, 10 (14%) (44% of all ICD patients) received an appropriate ICD shock and 19 (26%) died during a mean follow-up of 6.1±3.0 years. Thus, 29 patients (39%) reached the combined end-point. More events occurred in patients with ICM (n = 19) than in patients with DCM (n = 10) (59% vs. 24%; *p* = 0.002). Kaplan-Meier analysis was used to assess the prognostic value of APD_90_ restitution slope ≥1, APD_90_, and ERP/APD_90_ ratio. In patients with dual-site MAP recordings, only data from the RVA were used in the outcome analysis. Kaplan-Meier survival curves were calculated for the entire follow-up population and for the 2 subgroups of ICM and DCM patients, respectively. However, none of these parameters predicted outcome in any of the patient groups. Figure 3A shows Kaplan-Meier curves for event-free survival according to the presence or absence of steep restitution slope S_2_ (≥1). No significant differences were found for restitution slopes of S_2_ (*p* = 0.79; displayed in the graph), of S_3_ (*p* = 0.59), and of S_4_ (*p* = 0.38), respectively. APD_90_ did not predict outcome at BCL of 600, 500, 400 and 330 ms (*p* = 0.69, *p* = 0.45, *p* = 0.29 and *p* = 0.88, respectively). Figure 3B illustrates that ERP/APD_90_ ratio of S_1_ did not predict the combined end-point in the entire group (*p* = 0.57). Ratios of ERP/APD_90_ of S_2_ and S_3_ also were not predictive (*p* = 0.91 and *p* = 0.53, respectively). Subgroup analyses for ICM or DCM did not reveal any predictive value for APD_90_ restitution slope, APD_90_ or ERP/APD_90_ either.

Due to the limited number of microvolt TWA tests performed in our study, differences in combined end-point occurrence between patients with negative and non-negative test results could not be determined. In contrast, positive PVS was predictive of outcome in all patients (*p* = 0.006) (Figure 3C) and in patients with ICM (*p* = 0.03) but not in patients with DCM (*p* = 0.48).

## Discussion

### Main findings

To the best of our knowledge, this is the largest and longest follow-up study to investigate the prognostic implications of ventricular APD restitution slope characteristics in humans. Importantly, the study population included a significant number of patients with DCM. Moreover, it is the first study to relate ERP/APD ratios to long-term clinical outcome. We found that neither steepness of APD restitution slope nor ERP/APD ratios nor APD itself could predict long-term outcome in terms of mortality and/or appropriate ICD therapy in patients with severely impaired LV function due to ICM or DCM.

### Arrhythmia risk prediction in patients with cardiomyopathy

Identification of patients with cardiomyopathy who may benefit from ICD implantation is still based on impaired LV function [21]. Other contemporary risk stratifiers include TWA, ECG techniques such as signal-averaged ECG and heart rate variability, and testing of VT inducibility by means of PVS [22]. Our study reconfirmed the prognostic value of PVS with emphasis on the subgroup of ICM patients. In the subgroup of DCM patients we did not find PVS to be predictive of events. This is perfectly in line with earlier literature demonstrating the limited prognostic value of PVS in DCM patients, while a positive PVS is well described to identify patients with ICM who are at high risk of SCD [23], [24].

### Restitution slope – experimental studies

In basic EP studies, restitution slope measurements have provided a direct link to pathophysiology of malignant ventricular arrhythmias [9]. The original restitution hypothesis proposes that slopes <1 imply electrical stability with instability otherwise (slopes >1) [9], [25], [26]. An important link between APD restitution and arrhythmogenesis was suspected in the phenomenon of electrical alternans, either measured as APD alternans or TWA [27]. APD restitution slopes >1 may amplify alternans and ultimately lead to VF through electrical wavebreak in animal studies [6], [26]. Accordingly, flattening the APD restitution slope may dampen alternans and prevent VF [9]. Beside equivocal results of clinical studies on electrical restitution including our own study, criticism regarding the value of APD restitution slope as a predictor of VF has also been raised with regard to initial experimental concept [11], [12], [13], [28], [29]. Other mechanisms for alternans besides restitution and other mechanisms for VF besides alternans may be suspected and must exist if interpreting the clear-cut results of the current study [29]. Indeed, it should be remembered that the shape and therefore the slope of a given APD restitution curve are governed by complex interactions of various ion channels and that the restitution curve is just a part of a complex picture [27]. Intracellular Ca^2+^ cycling has been found to play a critical role in the development of APD alternans and wavebreak, independently of APD restitution kinetics [30]. The electrical restitution curve can further be modulated by myocardial ischemia, drugs, electrolyte shifts, and autonomic tone [27]. Most important, it has been shown that during ischemia the restitution curve is depressed and its slope is flattened [31], [32]. However, this ischemia-induced restitution slope flattening can hardly be regarded as physiologic or antiarrhythmic [27].

### Restitution slope in humans

The available human studies including our own data seem to extend the controversy established by experimental studies. Koller et al. investigated APD restitution slopes from a single RV recording site in 36 patients with and without structural heart disease and found similar slopes in both groups using a standard S_1_–S_2_ protocol and even higher values when employing a dynamic pacing protocol [12]. Dynamic pacing protocols were not used in our study to avoid the jeopardy of pacing at rates of >200 bpm in patients with severe ICM and DCM. This could also serve as an explanation why we did not observe APD alternans. The mean LVEF in the study of Koller et al. was markedly higher than in our study, thus the pathophysiology of the ventricular substrate may not be directly comparable [12]. Narayan et al. have recently reported that steep (≥1) APD restitution slopes, as determined by an S_1_–S_2_ protocol, can be observed both in control subjects and in patients with LVEF≤40% [13]. Moreover, APD restitution slope of S_2_≥1 was not related to TWA measurements and more importantly failed to predict outcome over a follow-up period of 2.3±1.3 years. Our results clearly confirm these findings and extend them considerably with respect to a longer follow-up duration (6.1±3.0 years) and a wider composition of the study population. We also characterized a significant number (n = 42) of patients with DCM with respect to APD restitution properties. Finally, we described restitution kinetics of additional extrastimuli (S_3_ and S_4_) for the first time. Clearly and in addition to the earlier results, we observed no differences between patients with ICM and DCM. The relatively low incidence of appropriate ICD therapy in our patients may be explained by the frequent administration of amiodarone. Our results also confirm the study of Narayan et al. in that no significant differences in restitution slope between RVA and the RVOT and between inducible and non-inducible patients were found [13]. This is in contrast to another study by Pak et al. [33]. These authors compared 10 inducible with 10 non-inducible patients at PVS and found a significantly higher APD restitution slope in inducible patients. However, the patient number in their study was low and no follow-up was reported.

Several clinical studies have attempted to assess restitution of repolarization by means of activation recovery intervals (ARI). Although being an adequate surrogate parameter of APD there may be profound methodological differences between ARI and APD measurements [34]. A study by Yue et al. has reported ARIs to be quite heterogeneous [35]. Nash et al. measured ARIs from 256 epicardial sites upon open cardiac surgery in 14 patients [36]. Both studies confirm that dispersion of restitution slopes obviously exists. None of them could however analyze a prognostic link. With regard to our own study and the study by Narayan et al., reproducibility of restitution slopes between the two crucial RV sites that PVS is performed from is still concordant with the mapping studies and there is no indication that such differences may obscure potential prognostic relevance [13]. Finally, Selvaraj et al. showed in 18 patients that maximum ARI restitution slopes are steeper in patients with positive TWA or inducible ventricular tachyarrhythmias [11]. Despite finding a significant difference, considerable overlap between restitution slope values of high-risk and low-risk patients existed. Aside from the above mentioned inherent inaccuracies of the ARI method, the definition of risk was solely based on inducibility and TWA which may not reflect the true risk encountered during subsequent follow-up.

### Prognostic value of APD and ERP/APD ratio in humans

To our knowledge, there is no published data to relate ERP/APD ratios or APD itself to prognosis. However, we did not find prognostic relevance of ERP/APD ratio or APD, nor a correlation to inducibility, despite successful prediction of prognosis by inducibility itself. In previous studies ERP/APD ratios have been investigated mainly to assess the actions of antiarrhythmic drugs [37]. Koller et al. found that the introduction of additional extrastimuli changes the fixed relation between ERP and APD, a finding which we could confirm [10]. In contrast, the ERP/APD ratio during steady-state pacing is constant and independent of BCL [38]. Small ERP/APD ratios have been associated with steep APD restitution slopes and inducibility of VT, however we did not find a correlation of these in our data [10], [11].

### Limitations

Our study has some limitations that deserve attention. Though it is the largest study of its kind, it is still a relatively small study evaluating 74 patients. This group size did not permit the separate analysis of end-points and larger studies are needed to confirm our findings. Another potential limitation of our study was the fact that all MAP recordings were performed at baseline conditions. Ischemia-induced changes of the restitution curve, which may alter the prognostic value of the restitution slope particularly in patients with ICM, were not evaluated. Furthermore, amiodarone therapy was initiated in several patients after PVS and MAP recordings had been performed. Therefore, we cannot exclude the possibility that the prognostic value of MAP derived parameters might have been influenced by amiodarone, but PVS remained predictive of the combined end-point under these conditions. Finally, for reasons of patient safety we recorded MAPs at only two RV sites. Future studies should record MAPs at multiple RV and LV sites to account for possible heterogeneities in APD restitution characteristics.

## Conclusions

As a stable result of this long-term clinical study, we found that neither APD_90_ nor ERP/APD_90_ nor APD_90_ restitution slope were predictive of prognosis or inducibility of VT at PVS.
